# PFKP deubiquitination and stabilization by USP5 activate aerobic glycolysis to promote triple-negative breast cancer progression

**DOI:** 10.1186/s13058-024-01767-z

**Published:** 2024-01-12

**Authors:** Zi-Mei Peng, Xiao-Jian Han, Tao Wang, Jian-Jun Li, Chun-Xi Yang, Fang-Fang Tou, Zhen Zhang

**Affiliations:** 1 Institute of Clinical Medicine, Jiangxi Provincial People’s Hospital, The First Affiliated Hospital of Nanchang Medical College, 152 Aiguo Road, Nanchang, 330006 Jiangxi China; 2 Institute of Geriatrics, Jiangxi Provincial People’s Hospital, The First Affiliated Hospital of Nanchang Medical College, Nanchang, Jiangxi China; 3 Department of Pulmonary and Critical Care Medicine, The First Affiliated Hospital of Soochow University, Suzhou, Jiangsu China; 4 Department of Oncology, Jiangxi Provincial People’s Hospital, The First Affiliated Hospital of Nanchang Medical College, Nanchang, Jiangxi China

**Keywords:** TNBC, USP5, PFKP, Aerobic glycolysis, Cancer progression

## Abstract

**Background:**

Triple-negative breast cancer (TNBC) remains the most challenging subtype of breast cancer and lacks definite treatment targets. Aerobic glycolysis is a hallmark of metabolic reprogramming that contributes to cancer progression. PFKP is a rate-limiting enzyme involved in aerobic glycolysis, which is overexpressed in various types of cancers. However, the underlying mechanisms and roles of the posttranslational modification of PFKP in TNBC remain unknown.

**Methods:**

To explore whether PFKP protein has a potential role in the progression of TNBC, protein levels of PFKP in TNBC and normal breast tissues were examined by CPTAC database analysis, immunohistochemistry staining (IHC), and western blotting assay. Further CCK-8 assay, colony formation assay, EDU incorporation assay, and tumor xenograft experiments were used to detect the effect of PFKP on TNBC progression. To clarify the role of the USP5-PFKP pathway in TNBC progression, ubiquitin assay, co-immunoprecipitation (Co-IP), mass spectrometry-based protein identification, western blotting assay, immunofluorescence microscopy, in vitro binding assay, and glycolysis assay were conducted.

**Results:**

Herein, we showed that PFKP protein was highly expressed in TNBC, which was associated with TNBC progression and poor prognosis of patients. In addition, we demonstrated that PFKP depletion significantly inhibited the TNBC progression in vitro and in vivo. Importantly, we identified that PFKP was a bona fide target of deubiquitinase USP5, and the USP5-mediated deubiquitination and stabilization of PFKP were essential for cancer cell aerobic glycolysis and TNBC progression. Moreover, we found a strong positive correlation between the expression of USP5 and PFKP in TNBC samples. Notably, the high expression of USP5 and PFKP was significantly correlated with poor clinical outcomes.

**Conclusions:**

Our study established the USP5-PFKP axis as an important regulatory mechanism of TNBC progression and provided a rationale for future therapeutic interventions in the treatment of TNBC.

**Supplementary Information:**

The online version contains supplementary material available at 10.1186/s13058-024-01767-z.

## Background

Breast cancer, one of the most common types of malignant tumors affecting women, has high incidence and mortality rates worldwide [1]. Breast cancer is a highly heterogeneous disease, which is molecularly classified into the luminal, HER2-overexpressing (HER2 +), and triple-negative subtypes. Triple-negative breast cancer (TNBC) is the most malignant subtype of breast cancer, which is defined as estrogen receptor (ER)-negative, progesterone receptor (PR)-negative, and human epidermal growth factor receptor type 2 (HER2)-negative disease [2]. Because of the lack of ER, PR, and HER2, TNBC is insensitive to established endocrine and anti-HER2 therapies, which carry a particularly unfavorable prognosis. Thus, there remains a great need to further explore the mechanisms of TNBC progression and identify potential therapeutic targets.

Aerobic glycolysis, also known as the Warburg effect, is among the most important mechanisms in tumor growth and progression [3]. During this process, cancer cells are characterized by increased glucose uptake and lactate production [4]. There are three important rate-limiting enzymes including hexokinase (HK), pyruvate kinase (PK), and phosphofructokinase (PFK) to control aerobic glycolysis. Human PFK exists as three isoforms: liver (PFKL), muscle (PFKM), and platelet (PFKP), which catalyzes the phosphorylation of fructose-6-phosphate to fructose-1, 6-bisphosphate during the glycolytic pathway [5]. PFKP is overexpressed in various types of cancers and its elevated expression is associated with poor prognosis. For example, PFKP alleviates glucose starvation-induced metabolic stress by regulating long-chain fatty acid oxidation via the AMPK-ACC2 axis in lung cancer [6]. In addition, PFKP levels are higher in hepatocellular carcinoma (HCC) as compared to normal hepatic tissues, which promotes HCC proliferation and contributes to the maintenance of HCC stemness [7]. A recent study in colorectal cancer also validates that PFKP is significantly overexpressed in cancer tissues and overexpression of PFKP contributes to the growth and invasion of cancer cells by regulating cell cycle progression [8]. However, the functions and mechanisms underlying PFKP expression in TNBC are still unclear, in particular the mechanisms regulating PFKP protein deubiquitination. Thus, further elucidating the underlying mechanisms of PFKP protein deubiquitination will identify new therapeutic strategies to deplete PFKP expression and develop personalized targeted therapies for TNBC.

Deubiquitinases (DUBs) are proteases of the ubiquitin–proteasome system that cleave ubiquitin from substrates as well as disassemble polyubiquitin chains [9]. Numerous pieces of evidence suggest that dysregulation of DUBs has been implicated in various human diseases, including cancer. For example, ATXN3 promotes prostate cancer progression by interacting with, deubiquitylating, and stabilizing YAP protein [10]. USP8 positively regulates hepatocellular carcinoma tumorigenesis and confers ferroptosis resistance through β-catenin stabilization [11]. In addition, USP13 regulates glycolytic reprogramming and progression in osteosarcoma by stabilizing METTL3/m6A/ATG5 axis [12]. Notably, growing evidence has indicated that several inhibitors of DUBs displayed potent anticancer activities. For example, the USP5 inhibitor EOAI prevents non-small cell lung cancer progression by inducing DNA damage [13]. And USP8 inhibitor DUB-IN-1 induces DNA damage, cell cycle arrest, apoptosis, and autophagy in esophageal squamous cell carcinoma [14]. Together, these studies indicate that DUBs are potential therapeutic targets in the treatment of cancer progression.

In the current study, we aim to investigate the expression and functions of PFKP in TNBC, furthermore, elucidate the underlying mechanisms of PFKP deubiquitination to identify new therapeutic strategies that deplete PFKP expression, and provide personalized targeted therapies for TNBC.

## Methods

### Database analysis

Protein expression data of PFKP in TNBC and normal samples from the Clinical Proteomic Tumor Analysis Consortium (CPTAC) cohort were analyzed based on the UALCAN (http://ualcan.path.uab.edu/) website [15]. The association between PFKP protein expression and the prognosis of TNBC patients was analyzed using the Kaplan–Meier plotter (KM plotter) database (http://kmplot.com/analysis/) [16]. The correlation between PFKP, deubiquitinase, and E3 ubiquitinase protein expression was analyzed by the LinkedOmics platform [17].

### Tissue microarray and IHC staining

A total of 172 cases of human TNBC and 130 cases of para-carcinoma tissues were obtained from Shanghai Superbiotek Pharmaceutical Technology Co., Ltd. The IHC staining and evaluation were performed as previously described [18], using 1:100 dilution of an anti-PFKP antibody (Santa Cruz, sc-514824) and 1:200 dilution of an anti-USP5 antibody (Proteintech, 10,473-1-AP).

### Cell culture

MDA-MB-231, SUM-159, and 293T Cell lines information and cell culture conditions were described previously [18]. The MCF-10A cell line was obtained from the American Type Culture Collection and cultured with DMEM/F12 medium supplemented with 5% horse serum, 100 U/mL penicillin, 100 mg/mL streptomycin, 20 ng/mL EGF, 0.5 mg/mL hydrocortisone, and 100 ng/mL cholera toxin in a humidified incubator at 37 °C with 5% CO_2_.

### Western blotting

Western blotting assay was performed as described previously [19], using 1:1000 dilution of an anti-PFKP antibody (Santa Cruz, sc-514824), 1:1000 dilution of an anti-USP5 antibody (Proteintech, 10,473-1-AP), and 1:1000 dilution of an anti-β actin antibody (Santa Cruz, sc-47778).

### RNA extraction and quantitative RT-PCR

RNA extraction and quantitative RT-PCR were performed as previously described [18]. β-actin was used as a normalization control. The primer sequences are listed in Additional file 1: Table S1.

### Lentiviral overexpression and knockdown systems

Lentiviral systems for overexpression of PFKP and knockdown of PFKP and USP5 were performed as described previously [18]. The primer sequences of PFKP and USP5 are listed in Additional file 1: Table S1.

### Cell proliferation assay

Cell proliferation was detected with CCK-8, colony formation, and EDU incorporation assays, which were performed as described previously [20].

### Tumor xenograft experiments

All experiments with mice were approved by the Ethics Committee at the Jiangxi Provincial People's Hospital. Six-week-old female BALB/c mice were purchased from Beijing Vital River Laboratory Animal Technology Co., Ltd. MDA-MB-231 cells (2 × 10^6^ cells in 100μL PBS) were injected into the fourth mammary fat pads of mice. The tumors were measured every 3 days after sizeable tumor formation. Then, the mice were euthanized, and tumors were dissected, weighed, and photographed after 20 days.

### Ubiquitination assay

Cells were treated with the proteasome inhibitor MG132 for 6 h before cell lysis. The cell lysates were prepared in RIPA buffer and incubated with an anti-PFKP antibody or IgG at 4 °C overnight. The immunoprecipitates were then used in a Western blotting assay with 1:1000 dilution of anti-ubiquitin antibody (Santa Cruz, sc-8017).

### Co-immunoprecipitation assay

Co-immunoprecipitation assay was performed as described previously [18], using 2 µg of anti-PFKP antibody (Santa Cruz, sc-514824) and 3 µg of anti-USP5 antibody (Proteintech, 10,473-1-AP) per 500 µg of total protein.

### Immunofluorescence microscopy

Immunofluorescence microscopy was performed as described previously [21], using 1:200 dilution of an anti-PFKP antibody (Santa Cruz, sc-514824) and 1:200 dilution of an anti-USP5 antibody (Proteintech, 10,473–1-AP).

### In vitro* binding assay*

Purified His-USP5 (MCE, HY-P74479) was incubated with purified GST-PFKP (Novus, H00005214-P01) or GST-Tag protein (Novus, NBC1-18,537) at 4 °C overnight. Then, the Glutathione Sepharose beads were added and incubated for 4 h. The bound proteins were then eluted in boiling 2 × SDS sample buffer and used in western blotting analysis.

### Glucose uptake, pyruvate, lactate, and ATP measurements

Glucose uptake was quantified using a commercially available Glucose Uptake Assay Kit (Abcam, ab136955). MDA-MB-231 and SUM-159 cells were seeded in six-well plates at a density of 3 × 10^5^ cells per well and incubated at 37 °C for 24 h. Prior to the assay, cells were subjected to serum deprivation to enhance glucose uptake. Subsequently, the cells were washed thrice with PBS buffer and starved in Krebs–Ringer-Phosphate-HEPES buffer for 40 min. Following this, the cells were stimulated with 100 nM insulin for 30 min. Finally, glucose uptake was assessed according to the manufacturer's instructions, with all results being normalized to cell number.

The Pyruvate Assay Kit (Abcam, ab65342) and Lactate Assay Kit (Abcam, ab65331) were utilized to perform the pyruvate and lactate assay, respectively. MDA-MB-231 and SUM-159 cells were seeded in six-well plates at a density of 3 × 10^5^ cells per well and incubated at 37 °C for 24 h. Subsequently, the cells were homogenized using the lysis buffer provided in each respective assay kit. The processed samples were then centrifuged, and the resulting supernatants were collected in fresh tubes. Finally, the measurement of pyruvate and lactate production was conducted according to the manufacturer’s instructions, with all results being normalized to the number of cells.

The ATP assay was conducted utilizing an ATP Assay Kit (Abcam, ab83355). Briefly, MDA-MB-231 and SUM-159 cells (1 × 10^6^ cells) were homogenized in ATP assay buffer and subjected to deproteinization using a Deproteinization Sample Preparation Kit (Abcam, ab284939). Ultimately, the ATP levels were quantified in accordance with the guidelines provided by the manufacturer. All outcomes were adjusted based on the number of cells.

### Extracellular acidification rate (ECAR) assays

The extracellular acidification rate (ECAR) was quantified utilizing the Seahorse XF24 Extracellular Flux Analyzer (Agilent Technologies). Experimental procedures were conducted in accordance with the guidelines provided by the manufacturer. Specifically, MDA-MB-231 and SUM-159 cells were seeded in Seahorse XF24 cell plates at a density of 2 × 10^4^ cells per well and incubated at 37 °C for 24 h. Prior to the assay, the cell plates were placed in a non-CO2 incubator for 30 min. Subsequently, the ECAR of the cells was assessed following the manufacturer's instructions. In the experimental procedure, glucose (10 mM), oligomycin (1 μM), and 2-DG (50 mM) were introduced into the wells in a sequential manner following each cycle. The resulting data were expressed as extracellular acidification rate (ECAR) normalized to protein concentration.

### Statistical analysis

Data were analyzed using GraphPad Prism 9 (GraphPad Software Inc) and SPSS-22 (SPSS Inc). The data from all of the experiments were presented as the means ± SEM. Spearman’s rank correlation test was used to analyze the correlation of gene expression in tissue samples. Student’s *t*-test for two groups was applied. The survival analysis was conducted using the Kaplan–Meier and log-rank tests. A *P*-value < 0.05 was considered significant. The *r*-value was used to evaluate the correlation analysis.

## Results

### PFKP protein expression is frequently upregulated in TNBC and is associated with poor prognosis in TNBC patients

**Fig. 1:**
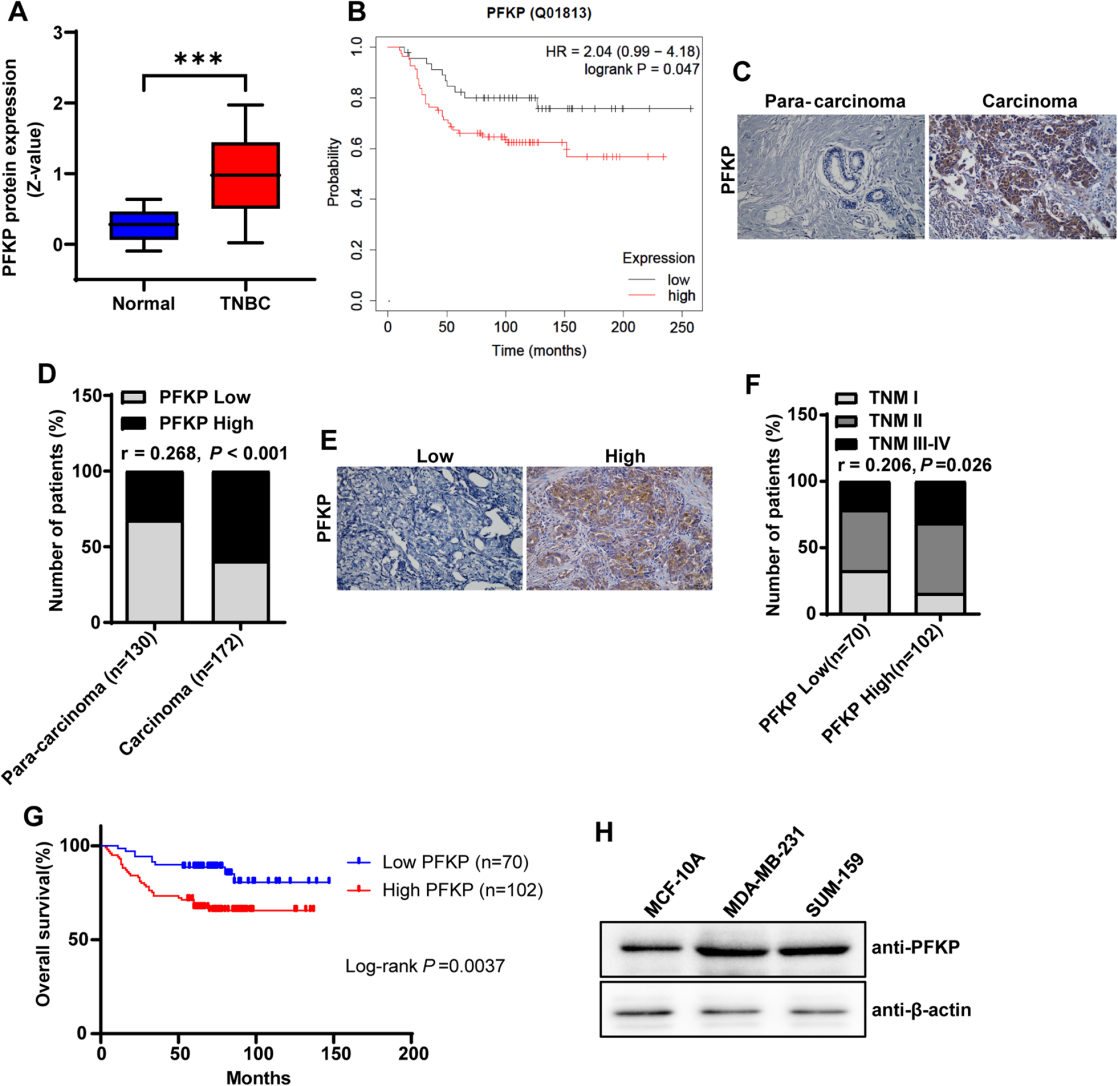
PFKP protein expression is frequently upregulated in TNBC and is associated with poor prognosis in TNBC patients. **a** The protein expression of PFKP is higher in TNBC than in normal breast tissues in CPTAC data. ***P < 0.001. **b** The survival curves of TNBC patients with high or low expression of PFKP protein based on the Kaplan–Meier plotter platform. **c** Representative images of IHC staining of PFKP in TNBC carcinoma and para-carcinoma tissues. Scale bars, 100 μm. **d** PFKP protein expression in TNBC carcinoma tissues (*n* = 172) was significantly higher than that in para-carcinoma tissues (*n* = 130). *r* = 0.268, *P* < 0.001. **e** Representative images of IHC staining of PFKP in TNBC carcinoma tissues. Scale bars, 100 μm. **f** The expression of PFKP correlates positively with the TNM stage in human TNBC carcinoma samples. *r* = 0.206, *P* = 0.026. **g** High expression of PFKP is correlated with poor overall survival of TNBC patients. Log-rank *P* = 0.0037. **h** The protein expression of PFKP in TNBC cell lines (MDA-MB-231 and SUM-159) and normal breast cell line (MCF-10A)

To explore whether PFKP protein has a potential role in the progression of TNBC, we queried the Clinical Proteomic Tumor Analysis Consortium (CPTAC) datasets using the UALCAN website. The results of the analysis showed that PFKP protein expression was significantly higher in TNBC samples compared with normal breast samples (Fig. 1a). Notably, survival analysis based on the KM plotter database showed a negative correlation of PFKP protein expression with overall survival in TNBC patients (Fig. 1b). We further detected the expression of PFKP in TNBC carcinoma and para-carcinoma tissues by immunohistochemistry, and the results showed that the protein expression level of PFKP was higher in carcinoma tissues than that in para-carcinoma tissues in patients with TNBC (Fig. 1c, d). Then, the TNBC carcinoma tissue samples were stratified into two groups, according to high or low expression of the PFKP protein (Fig. 1e). As shown in Fig. 1f, the protein expression level of PFKP was positively associated with the TNM stage in TNBC patients. Moreover, immunohistochemical staining showed that the patients with high PFKP expression exhibited poorer overall survival compared with patients with low PFKP expression (Fig. 1g). In addition, we further interrogated the PFKP protein expression in TNBC cell lines (MDA-MB-231 and SUM-159) and normal breast cell lines (MCF-10A) and identified the level of PFKP protein was higher in TNBC cell lines compared with normal breast cell line (Fig. 1h). Together these results demonstrated that PFKP protein expression was frequently upregulated in TNBC and was associated with poor prognosis in TNBC patients.

### *Inhibition of PFKP reduces TNBC progression *in vitro* and *in vivo

**Fig. 2:**
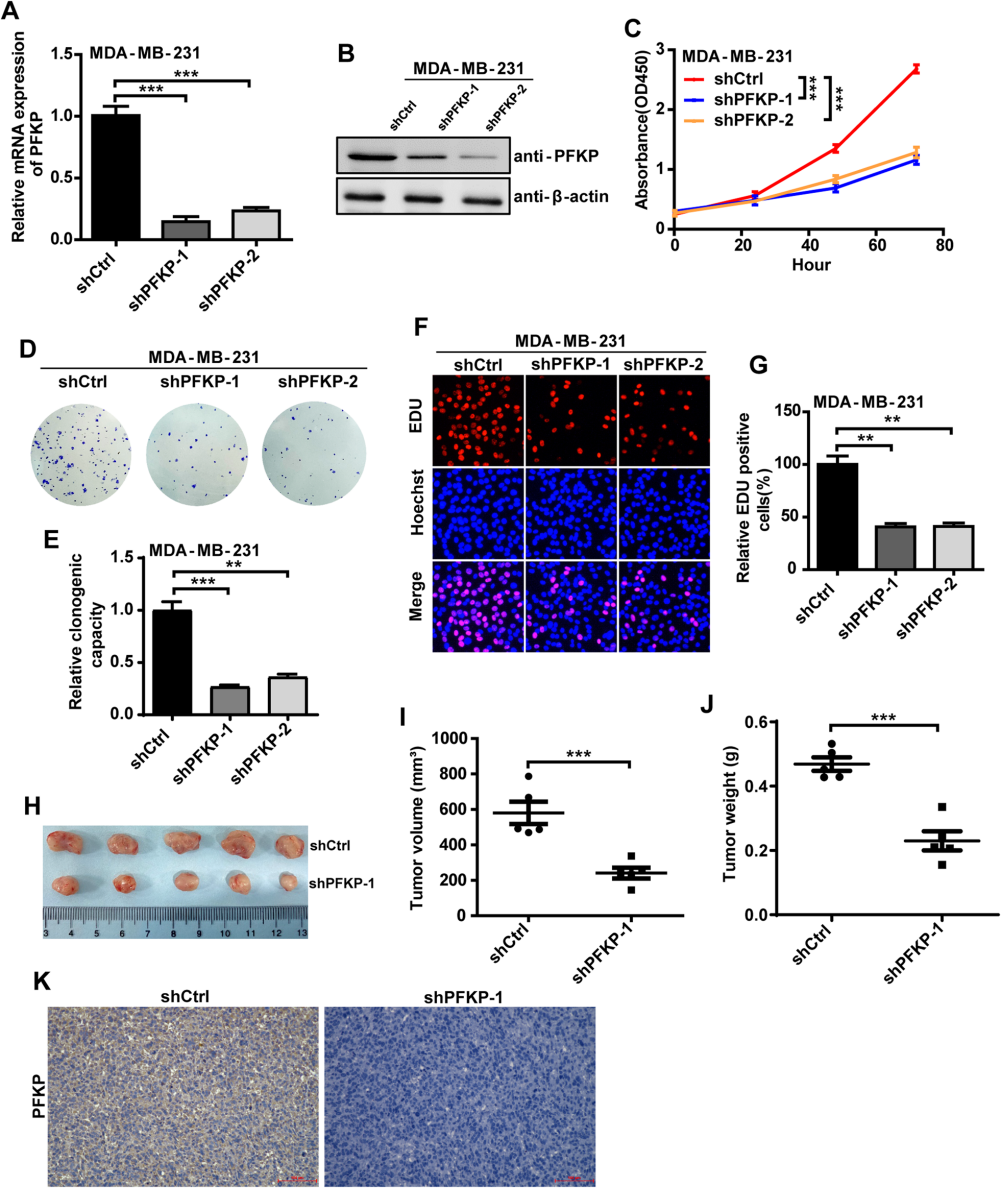
Inhibition of PFKP reduces TNBC progression in *vitro* and in vivo. **a**, **b** qRT-PCR (**a**) and western blot (**b**) analysis of PFKP expression with PFKP knockdown in MDA-MB-231 cells. ***P < 0.001. **c** CCK-8 assay determination of MDA-MB-231 cell proliferation in response to PFKP knockdown. ***P < 0.001. **d**, **e** Clone formation assay determination of MDA-MB-231 cell proliferation in response to PFKP knockdown. ***P < 0.001, **P < 0.01. **f**, **g** EDU incorporation assay determination of MDA-MB-231 cell proliferation in response to PFKP knockdown. **P < 0.01. **h** Representative images of the xenograft tumors in each group. **i**, **j** PFKP knockdown reduces tumor volume (**i**) and tumor weight (**j**) of nude mice. ***P < 0.001. **k** The PFKP protein expression in each sample of mice tumor tissue was analyzed by IHC staining. Scale bars, 100 μm

To further confirm the role of PFKP in TNBC progression, we used lentiviral shRNA to knockdown PFKP in MDA-MB-231 and SUM-159 cells and found that the shRNAs specific for PFKP efficiently downregulated PFKP expression (Fig. 2a, b and Additional file 1: Figure S1A, S1B). Then, we examined the effect of PFKP on TNBC progression by CCK-8 assay, colony-formation assay, and EDU incorporation assay in MDA-MB-231 and SUM-159 cells. The results showed that inhibition of PFKP significantly reduced TNBC progression in vitro (Fig. 2c–g and Additional file 1: Figure S1C–S1G). To further determine the role of PFKP on TNBC progression in vivo, we established xenograft tumor models in nude mice. To do so, MDA-MB-231 cells stably expressing control shRNA or PFKP shRNA were implanted into the mammary fat pad of female BALB/c nude mice, and tumor growth was examined. The results showed that PFKP inhibition significantly suppressed tumor growth, tumor volume, and tumor weight (Fig. 2h–j). Moreover, an immunohistochemistry staining assay was used to investigate the expression of PFKP protein in each sample of mice tumor tissue. As shown in Fig. 2k, PFKP protein expression was significantly decreased in PFKP-knockdown tumors. Collectively, these results suggested that PFKP played a key role in TNBC progression and the inhibition of PFKP was an effective strategy to curb TNBC progression.

### USP5 is a bona fide deubiquitinase that targets PFKP protein for deubiquitination and stabilization in TNBC

**Fig. 3:**
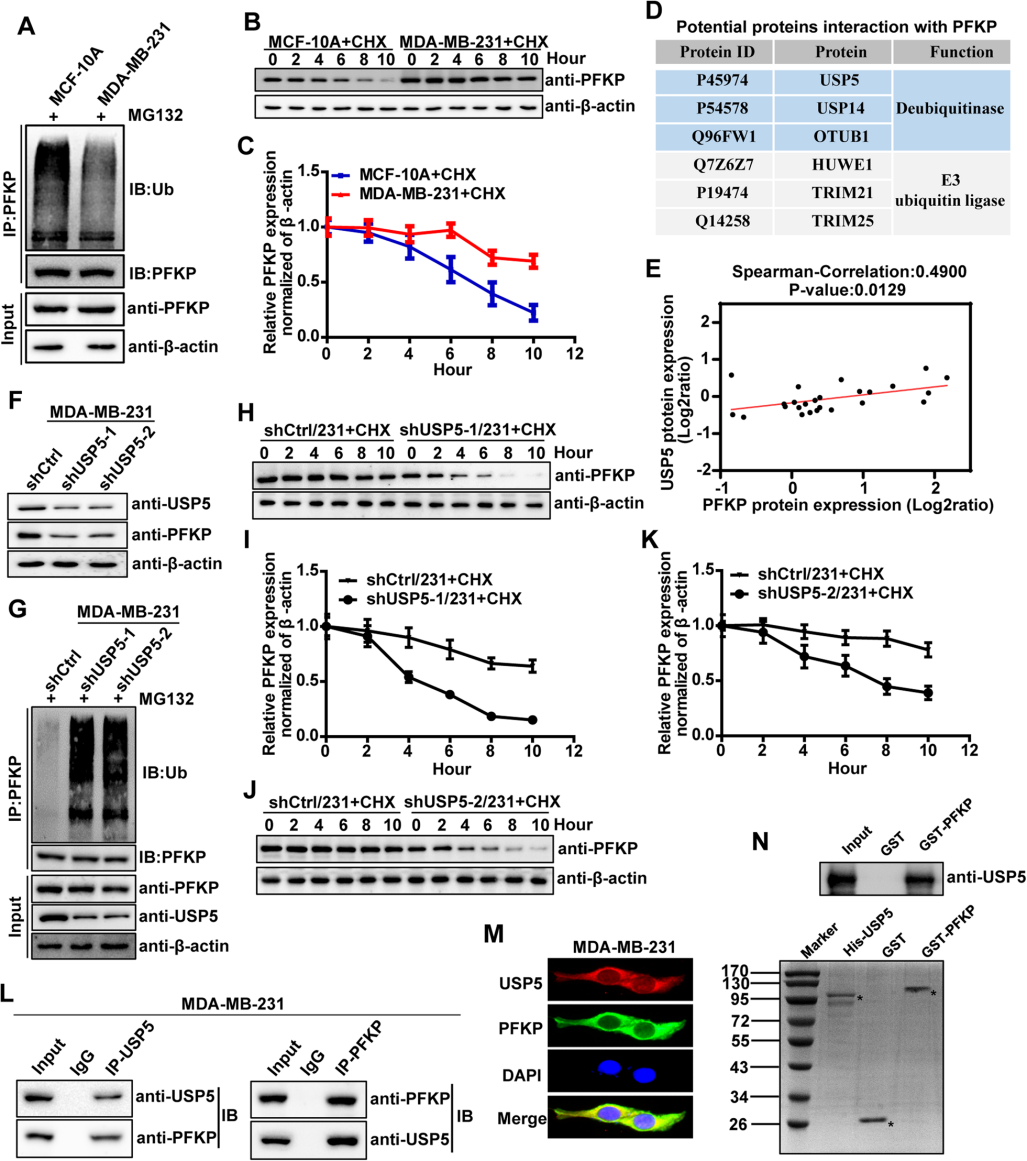
USP5 is a bona fide deubiquitinase that targets PFKP protein for deubiquitination and stabilization in TNBC. **a** Co-IP analysis of PFKP protein ubiquitination in MCF-10A and MDA-MB-231 cells. **b** MCF-10A and MDA-MB-231 cells were treated with CHX for the indicated time points and analyzed by Western blot. **c** Quantification of PFKP protein levels normalized to β-actin in time after the addition of CHX. **d** The diagram showing three deubiquitinases (USP5, USP14, and OTUB1) and three E3 ubiquitin ligases (HUWE1, TRIM21, and TRIM25) could interact with PFKP identified by a combination of Co-IP and MS. **e** The correlation between USP5 protein expression and PFKP protein expression in TNBC was analyzed by LinkedOmics platform. **f** The USP5 and PFKP protein expression in control and USP5 knockdown MDA-MB-231 cells were analyzed by Western blot. **g** Co-IP analysis of PFKP protein ubiquitination in control and USP5 knockdown MDA-MB-231 cells. **h–k** Control and USP5 knockdown MDA-MB-231 cells were treated with CHX for the indicated time points and analyzed by Western blot **h**, **j**, quantification of PFKP protein levels normalized to β-actin in time after the addition of CHX (**i**, **k**). **l** The binding of USP5 to PFKP was detected by Co-IP assay in MDA-MB-231 cells. **m** Immunofluorescence co-localization of USP5 and PFKP in MDA-MB-231 cells. **n** In vitro binding assays of purified GST-PFKP and His-USP5 proteins

To explore the underlying mechanisms involved in the regulation of PFKP protein expression in TNBC, we performed an ubiquitin assay to detect the ubiquitination level of PFKP protein in TNBC cell line MDA-MB-231 and normal breast cell line MCF-10A. As shown in Fig. 3a, the ubiquitination level of PFKP protein in MDA-MB-231 cells was significantly lower than that in MCF-10A cells. In line, the PFKP protein stability (half-life) was significantly increased in MDA-MB-231 cells in response to the protein synthesis inhibitor cycloheximide (CHX) treatment, compared to that in MCF-10A cells (Fig. 3b, c). Together, these observations demonstrated that high expression of PFKP protein in TNBC might be regulated by the posttranslational modification: ubiquitination-mediated degradation.

Given that the E3 ubiquitin ligases and deubiquitinases play central roles in protein degradation and turnover through protein ubiquitination and deubiquitination [22], we performed the Co-immunoprecipitation (Co-IP) and mass spectrometry-based protein identification, to identify E3 ubiquitin ligases and deubiquitinases that interacted with PFKP. The results showed that three deubiquitinases (USP5, USP14, and OTUB1) and three E3 ubiquitin ligases (HUWE1, TRIM21, and TRIM25) could potentially interact with PFKP in MDA-MB-231 cells (Fig. 3d). Notably, the correlation between USP5 protein and PFKP protein expression levels was much higher as compared with the five other E3 ubiquitin ligases and deubiquitinases in TNBC (Fig. 3e and Additional file 1: Figure S2A-2E), indicating that USP5 might regulate PFKP protein deubiquitination. To substantiate this finding, we performed the immunoblotting and ubiquitination assay in MDA-MB-231 and SUM-159 cells with or without USP5 knockdown. The results showed that the knockdown of USP5 by specific shRNAs decreased PFKP protein expression while increasing the ubiquitination level of PFKP protein (Fig. 3f, g and Additional file 1: Figure S2F, S2G). In addition, USP5 depletion promoted PFKP protein degradation in response to treatment with CHX in MDA-MB-231 and SUM-159 cells (Fig. 3h–k and Additional file 1: Figure S2H-S2K). Furthermore, we performed a Co-IP assay and immunofluorescence assay to investigate the interaction of USP5 and PFKP proteins in MDA-MB-231 and SUM-159 cells. The results of the Co-IP assay showed that USP5 and PFKP proteins were bound to each other (Fig. 3l and Additional file 1: Figure S2L), and the immunofluorescence assay indicated that USP5 co-localized with PFKP proteins in the cytoplasm (Fig. 3m and Additional file 1: Figure S2M). Notably, an in vitro binding assay proved that the purified proteins of His-USP5 and GST-PFKP could directly bind to each other under cell-free conditions (Fig. 3n). Collectively, our data demonstrated that USP5 was a bona fide deubiquitinase that directly targeted PFKP protein for deubiquitination and stabilization in TNBC.

### USP5 regulates aerobic glycolysis in TNBC

**Fig. 4:**
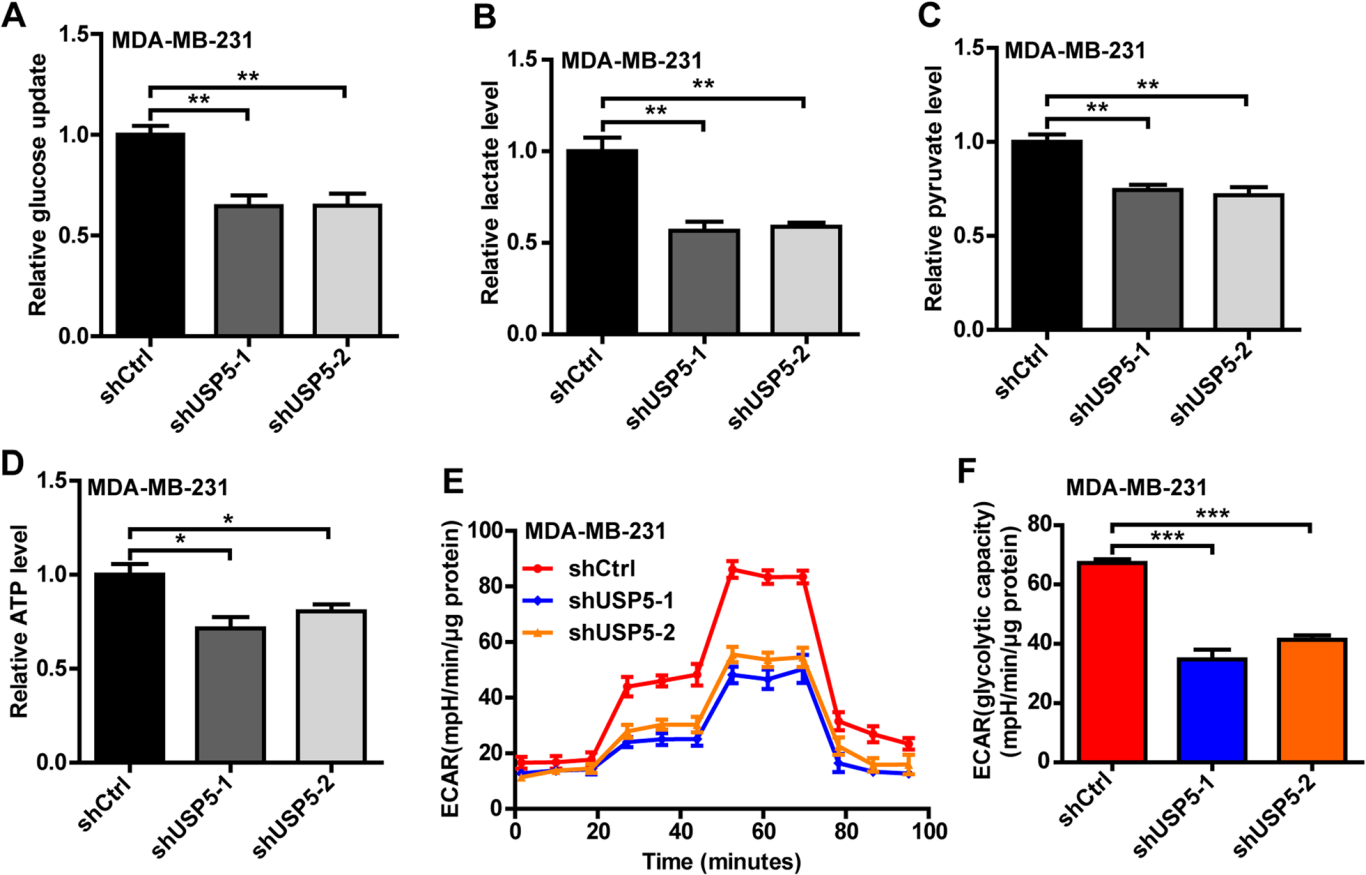
USP5 regulates aerobic glycolysis in TNBC. **a**–**d** Glucose update (**a**), lactate level (**b**), pyruvate level (**c**), and ATP level (**d**) were significantly decreased after USP5 knockdown in MDA-MB-231 cells. **e**, **f** ECAR values (**e**) and calculated glycolytic capacity (**f**) of control and USP5 knockdown MDA-MB-231 cells

Aerobic glycolysis is a common feature of glucose metabolism in cancer cells, and PFKP is an important rate-limiting enzyme that regulates aerobic glycolysis. Given that our study demonstrated that USP5 deubiquitinated and stabilized PFKP protein in TNBC, we next explore whether USP5 regulates aerobic glycolysis in TNBC. As shown in Fig. 4a–d and Additional file 1: Figure S3A—S3D, USP5 depletion reduced the glucose uptake, lactate level, pyruvate level, and ATP level in MDA-MB-231 and SUM-159 cells. Moreover, the potential regulatory role of USP5 in TNBC glucose metabolism was further explored by ECAR assay, and results showed that the knockdown of USP5 significantly reduced ECAR and glycolytic capacity (Fig. 4e, f and Additional file 1: Figure S3E, S3F). Taken together, our results demonstrated that USP5 regulated aerobic glycolysis in TNBC cells.

### *USP5 promotes TNBC progression by regulating PFKP protein expression *in vitro* and *in vivo

**Fig. 5:**
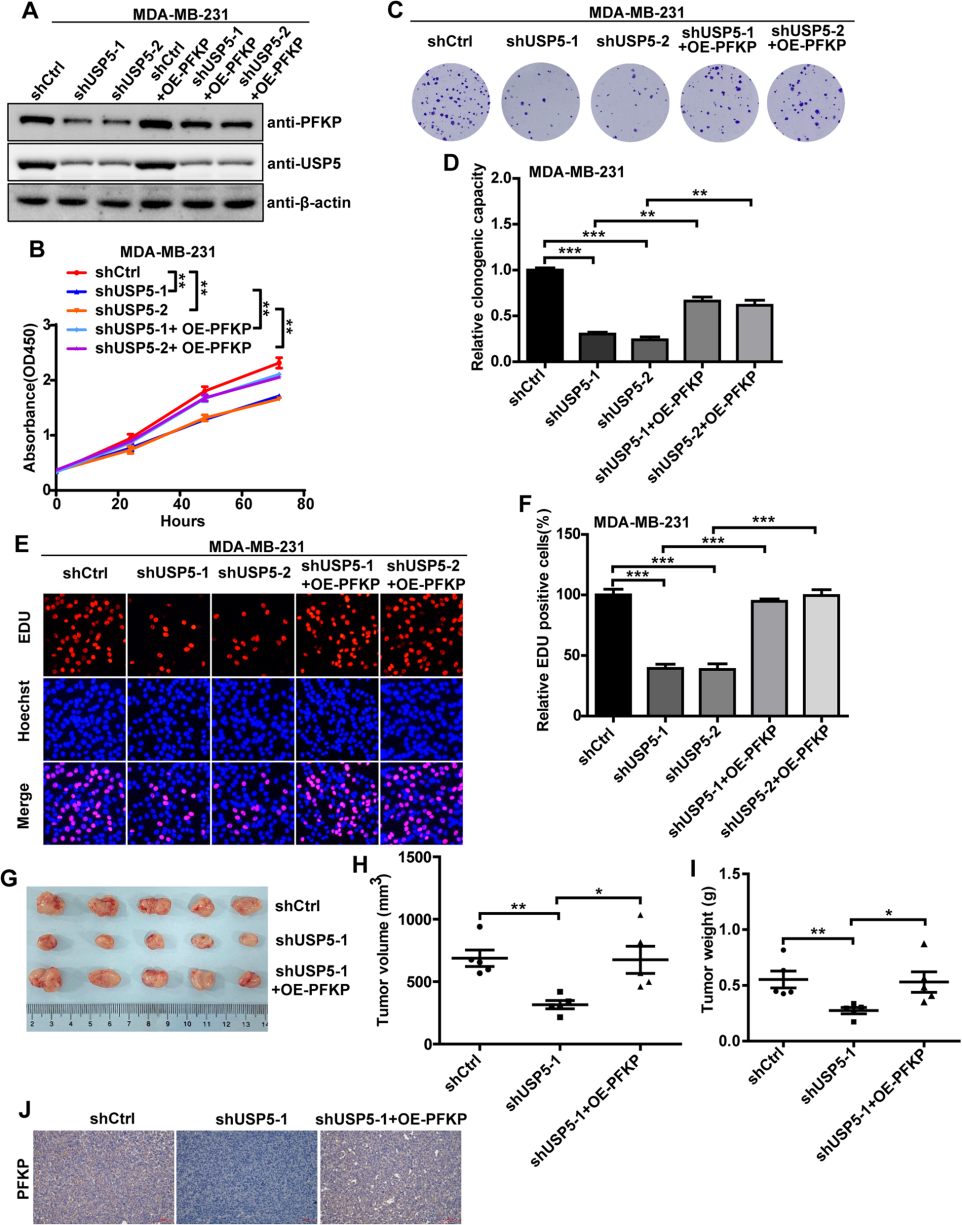
USP5 promotes TNBC progression by regulating PFKP protein expression in vitro and in vivo. **a** The USP5 and PFKP protein expression in control and USP5 knockdown MDA-MB-231 cells in the presence or absence of overexpressed PFKP were analyzed by Western blot. **b–f** MDA-MB-231 cell proliferation following USP5 knockdown in the presence or absence of overexpressed PFKP was evaluated using CCK8 assay (**b**), Clone formation assay (**c**, **d**), and EDU incorporation assay (**e**, **f**). ***P < 0.001, **P < 0.01. **g** Representative images of the xenograft tumors in each group. **h**, **i** Tumor volume (**h**) and tumor weight (**i**) of nude mice in each group. **P < 0.01, *P < 0.05. **j** The PFKP protein expression in each sample of mice tumor tissue was analyzed by IHC staining. Scale bars, 100 μm

Given that USP5 deubiquitinates and stabilizes PFKP protein in TNBC, we predict whether USP5 promotes TNBC progression by regulating PFKP protein expression. To do so, PFKP was overexpressed based on USP5 knockdown in TNBC cells. The immunoblotting assay showed that the decrease of PFKP caused by the USP5 knockdown could be rescued by exogenous overexpression of PFKP (Fig. 5a and Additional file 1: Figure S4A). Next, CCK-8 assay, colony-formation assay, and EDU incorporation assay showed that USP5 depletion significantly inhibited the proliferation of MDA-MB-231 and SUM-159 cells in vitro, while this inhibitory effect was reversed by exogenous overexpression of PFKP (Fig. 5b–f and Additional file 1: Figure S4B-S4F). In line, the xenograft nude mice model was applied to determine whether USP5 promotes TNBC progression by regulating PFKP protein expression in vivo. And we found that the knockdown of USP5 significantly decreased the growth, volume, weight, and PFKP protein expression of tumors in mice; however, this effect was attenuated in mice carrying PFKP-overexpressing tumors (Fig. 5g–j), confirming that the knockdown of USP5 reduces TNBC progression through the regulation of PFKP in vivo. Collectively, these results revealed that USP5 promoted TNBC progression by regulating PFKP protein expression in vitro and in vivo.

### USP5 is positively correlated with PFKP expression in TNBC and is associated with poor prognosis in TNBC patients

**Fig. 6:**
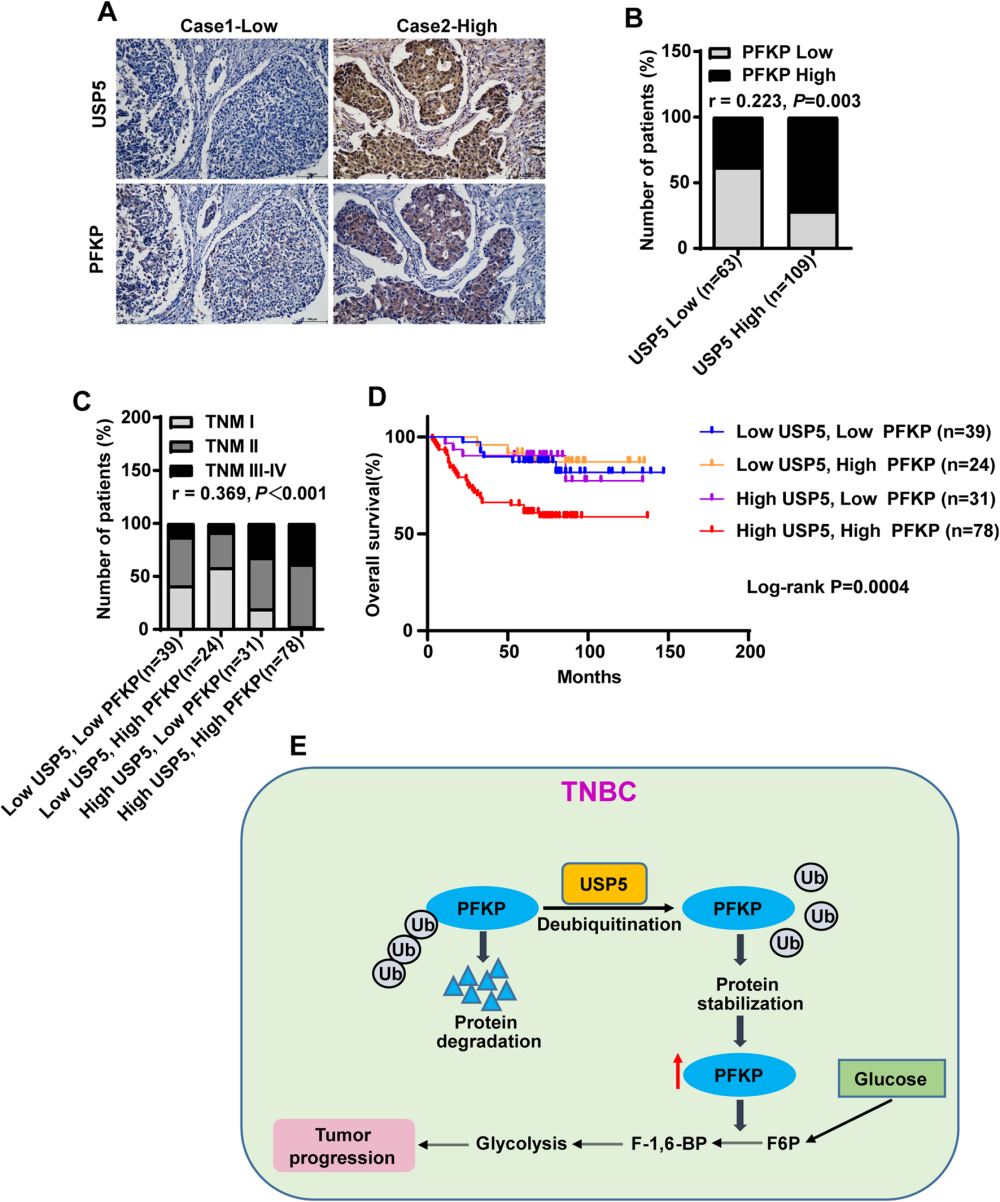
USP5 is positively correlated with PFKP expression in TNBC and is associated with poor prognosis of TNBC patients. **a** Representative images of IHC staining of USP5 and PFKP in serial sections of the same TNBC tumor. Scale bars, 100 μm. **b** A positive correlation between the expression of USP5 and PFKP in the 172 human TNBC carcinoma samples. *r* = 0.223, *P* = 0.003. **c** Concomitantly high expression of USP5 and PFKP correlates positively with the TNM stage in human TNBC carcinoma samples. *r* = 0.369, *P* < 0.001. **d** Kaplan–Meier curves showed that the overall survival of patients with concomitantly high USP5 and PFKP expression in TNBC tumors was shorter than that of those with concomitantly low USP5 and PFKP expression. Log-rank *P* = 0.0004. **e** A schematic diagram of USP5 promotes TNBC progression and aerobic glycolysis through deubiquitinating and stabilizing PFKP

To confirm the role of the USP5-PFKP signaling pathway in TNBC progression, we performed immunohistochemistry to detect the expression of USP5 and PFKP in the 172 cases of human TNBC carcinoma tissues and analyzed the correlations between USP5 and PFKP in TNBC tissues. The results indicated a strong positive correlation between the expression of USP5 and PFKP (Fig. 6a, b). Importantly, we demonstrated concomitantly high expression of USP5 and PFKP in TNBC patients with advanced TNM stage (Fig. 6c), highlighting that dysregulated USP5-PFKP signaling was functionally linked to cancer progression in TNBC. In addition, the survival analysis indicated that TNBC patients with concomitantly high expression of USP5 and PFKP in their tumors had shorter overall survival than those with low USP5 and PFKP expression (Fig. 6d). Altogether, these results revealed that aberrant functionality of the USP5-PFKP axis might contribute to TNBC progression and could be used to predict poor clinical outcomes in TNBC patients.

## Discussion

Although an increase in PFKP expression is a characteristic feature of malignant tissues, little is known about how PFKP expression is regulated during the development and progression of cancers. Only a handful of studies have explored the molecular mechanisms changing the expression of PFKP in cancer. For example, at the transcriptional level, BRCA1/ZBRK1 complex and Snail restrain PFKP expression [23, 24], while KLF4 increases the expression of PFKP by directly binding to the PFKP promoter [25]. In addition, at the posttranslational level, E3 ubiquitin ligase HRD1 and TRIM21 interact and target PFKP for ubiquitination and degradation, which ultimately reduces PFKP expression [26, 27]. However, the mechanisms regulating PFKP protein deubiquitination, a process opposing ubiquitination, remain unexplored. Here, we identified that PFKP was a bona fide target of deubiquitinase USP5 and USP5 directly deubiquitinated and stabilized PFKP in TNBC, which provided the new therapeutic strategies to deplete PFKP expression and to develop personalized targeted therapies of TNBC. Notably, besides USP5, based on Co-immunoprecipitation (Co-IP)/mass spectrometry-based protein identification and correlation analysis (Fig. 3d and Additional file 1: Figure S2A), our study also showed that another deubiquitinase USP14 was a potential deubiquitinating enzyme of PFKP, but whether it regulates PFKP protein deubiquitination and stabilization needs to be further validated in future studies.

PFKP is a significant enzyme that serves as a rate-limiting factor in the regulation of aerobic glycolysis. Our study provides evidence that USP5 plays a role in deubiquitinating and stabilizing the PFKP protein in TNBC, prompting us to investigate the impact of USP5 on the regulation of aerobic glycolysis in TNBC. Consistent with our expectations, we observed that depletion of USP5 effectively suppressed aerobic glycolysis in TNBC cells. It is worth noting that previous research has shown that USP5 also targets the Hypoxia-inducible factor (HIF) protein [28], which is known to regulate aerobic glycolysis [29]. Thus, it is hypothesized that USP5 plays a role in the regulation of aerobic glycolysis through its deubiquitinating and stabilizing effects on the PFKP protein, as well as its regulation of the HIF protein. Notably, a previous study has demonstrated that PFKP is a downstream target of the HIF protein [30]. Therefore, it can be inferred that USP5 influences breast cancer progression by targeting either the HIF protein [28] or the PFKP protein, which in turn may directly or indirectly impact the function of PFKP. This finding also provides an explanation for the significant rescue of TNBC progression inhibition observed upon exogenous overexpression of PFKP following USP5 knockdown in the present study. Furthermore, it is worth conducting further investigation to determine if other downstream targets of USP5 also play a role in regulating the function of PFKP.

USP5, also referred to as isopeptidase T (ISOT), is a deubiquitinase of the ubiquitin–proteasome system, which removes unanchored polyubiquitin chains on protein substrates and plays an essential role in several diseases [31]. Notably, a growing body of evidence suggests that USP5 is widely expressed in various cancers and contributes significantly to cancer progression. For example, USP5 protein is highly expressed in non-small cell lung cancer tissues and closely correlates with poor prognosis of these patients; and USP5 facilitates non-small cell lung cancer progression by directly interacting with, deubiquitinating, and stabilizing PD-L1 protein [32]. In hepatocellular carcinoma, USP5 interacts with and stabilizes SLUG to promote the EMT and malignant progression of cancer [33]. Consistent with these previous studies, our study showed that USP5 was positively correlated to PFKP in TNBC tissues, and concomitantly high expression of USP5 and PFKP were correlated with poor prognosis of TNBC patients. Moreover, the knockdown of USP5 significantly reduced TNBC progression through the regulation of PFKP, suggesting that USP5 may serve as a potential therapeutic target for patients with cancer.

Cancer cells reprogram their glucose metabolism that produce energy predominantly by glycolysis rather than by mitochondrial oxidative phosphorylation even under aerobic conditions, which is termed as Warburg effect or aerobic glycolysis [34]. In addition to providing cellular energy, the metabolic intermediates generated during aerobic glycolysis can be used for macromolecular biosynthesis, which facilitates the rapid growth of cancer cells [35]. Studies have shown that metabolic changes in cancer cells from cytoplasm-based glycolysis to mitochondria-based glucose oxidation can effectively decrease tumor growth [36], indicating that targeting aerobic glycolysis in cancer cells is a promising therapeutic strategy. Taken together with our results showed that knockdown of USP5 significantly inhibited the aerobic glycolysis process, further development of USP5 inhibitors to reverse the aerobic glycolysis of cancer cells and reduce cancer progression may contribute to future clinical treatment of cancer.

## Conclusions

In this study, we showed that PFKP protein was highly expressed in TNBC tissues and cells, which was associated with TNBC progression and poor prognosis. In addition, using a series of experiments, we demonstrated that PFKP depletion significantly inhibited the TNBC progression in vitro and in vivo. Importantly, we identified that PFKP was a bona fide target of deubiquitinase USP5, and the USP5-mediated deubiquitination and stabilization of PFKP were essential for cancer cell aerobic glycolysis and TNBC progression. Thus, our study established the USP5-PFKP axis as an important regulatory mechanism of TNBC progression and provided a rationale for future therapeutic interventions in the treatment of TNBC (Fig. 6e).

### Supplementary Information

**Additional file 1**. Supplementary Information of PFKP deubiquitination and stabilization by USP5 activate aerobic glycolysis to promote triple-negative breast cancer progression, Figures S1–S4 and Table S1.

## Data Availability

The data and materials of this study are available from the corresponding author upon reasonable request.

## Abbreviations

PFKP: ATP-dependent 6-phosphofructokinase, platelet type
USP5: Ubiquitin carboxyl-terminal hydrolase 5
TNBC: Triple-negative breast cancer
CPTAC: Clinical proteomic tumor analysis consortium
IHC: Immunohistochemistry
CCK-8: Cell counting Kit-8
EDU: 5-Ethynyl-2’-deoxyuridine
HER2: Human epidermal growth factor receptor type 2
ER: Estrogen receptor
PR: Progesterone receptor
HK: Hexokinase
PK: Pyruvate kinase
PFK: Phosphofructokinase
AMPK: AMP-activated protein kinase
ACC2: Acetyl-CoA carboxylase 2
HCC: Hepatocellular carcinoma
DUBs: Deubiquitinases
ATXN3: Ataxin-3
YAP: Yes-associated protein
USP8: Ubiquitin carboxyl-terminal hydrolase 8
USP13: Ubiquitin carboxyl-terminal hydrolase 13
METTL3: N6-adenosine-methyltransferase catalytic subunit
m6A: N6-methyladenosine
ATG5: Autophagy protein 5
ATP: Adenosine 5'-triphosphate
CHX: Cycloheximide
shRNAs: Small hairpin RNAs
ECAR: Extracellular acidification rate
TNM: Tumor node metastasis classification
USP14: Ubiquitin carboxyl-terminal hydrolase 14
OTUB1: OTU domain-containing ubiquitin aldehyde-binding protein 1
HUWE1: HECT, UBA and WWE domain-containing protein 1
TRIM21: Tripartite motif-containing protein 21
TRIM25: Tripartite motif-containing protein 25
BRCA1: Breast cancer type 1 susceptibility protein
ZBRK1: Zinc finger and BRCA1-interacting protein with a KRAB domain 1
KLF4: Kruppel-like factor 4
HRD1: HMG-CoA reductase degradation protein 1
HIF: Hypoxia-inducible factor
PD-L1: Programmed cell death 1 ligand 1
SLUG: Neural crest transcription factor Slug
EMT: Epithelial-mesenchymal transition
