# Supplementary material for: PFKP deubiquitination and stabilization by USP5 activate aerobic glycolysis to promote triple-negative breast cancer progression
Source: Breast Cancer Res. 2024 Jan 12;26:10. doi: 10.1186/s13058-024-01767-z (PMC10787506; doi:10.1186/s13058-024-01767-z)
Supplement: Supplementary file 1 — Additional file 1. Supplementary Information of PFKP deubiquitination and stabilization by USP5 activate aerobic glycolysis to promote triple-negative breast cancer progression, Figures S1–S4 and Table S1. [file 13058_2024_1767_MOESM1_ESM.pdf]

Supplementary Information for

PFKP deubiquitination and stabilization by USP5 activates aerobic glycolysis to promote triple-negative breast cancer progression

Zi-Mei Peng[1], Xiao-Jian Han[2], Tao Wang[2], Jian-Jun Li[3], Chun-Xi Yang[1], Fang-Fang Tou[4, *], Zhen Zhang[1, *]

[1]Institute of Clinical Medicine, Jiangxi Provincial People's Hospital, The First Affiliated Hospital of Nanchang Medical College, Nanchang, Jiangxi, China

[2]Institute of Geriatrics, Jiangxi Provincial People's Hospital, The First Affiliated Hospital of Nanchang Medical College, Nanchang, Jiangxi, China

[3]Department of Pulmonary and Critical Care Medicine, The First Affiliated Hospital of Soochow University, Suzhou, Jiangsu, China

[4]Department of Oncology, Jiangxi Provincial People's Hospital, The First Affiliated Hospital of Nanchang Medical College, Nanchang, Jiangxi, China

*Corresponding author: Zhen Zhang
Jiangxi Provincial People's Hospital, The First Affiliated Hospital of Nanchang Medical College, 152 Aiguo Road, Nanchang 330006, Jiangxi, China
E-mail: nkzhenzhang@163.com

Fang-Fang Tou
Jiangxi Provincial People's Hospital, The First Affiliated Hospital of Nanchang Medical College, 152 Aiguo Road, Nanchang 330006, Jiangxi, China
E-mail: toufangfang@163.com

This file includes: Supplementary Fig. S1-S4 and Supplementary Tables S1.

Sup Figure 1

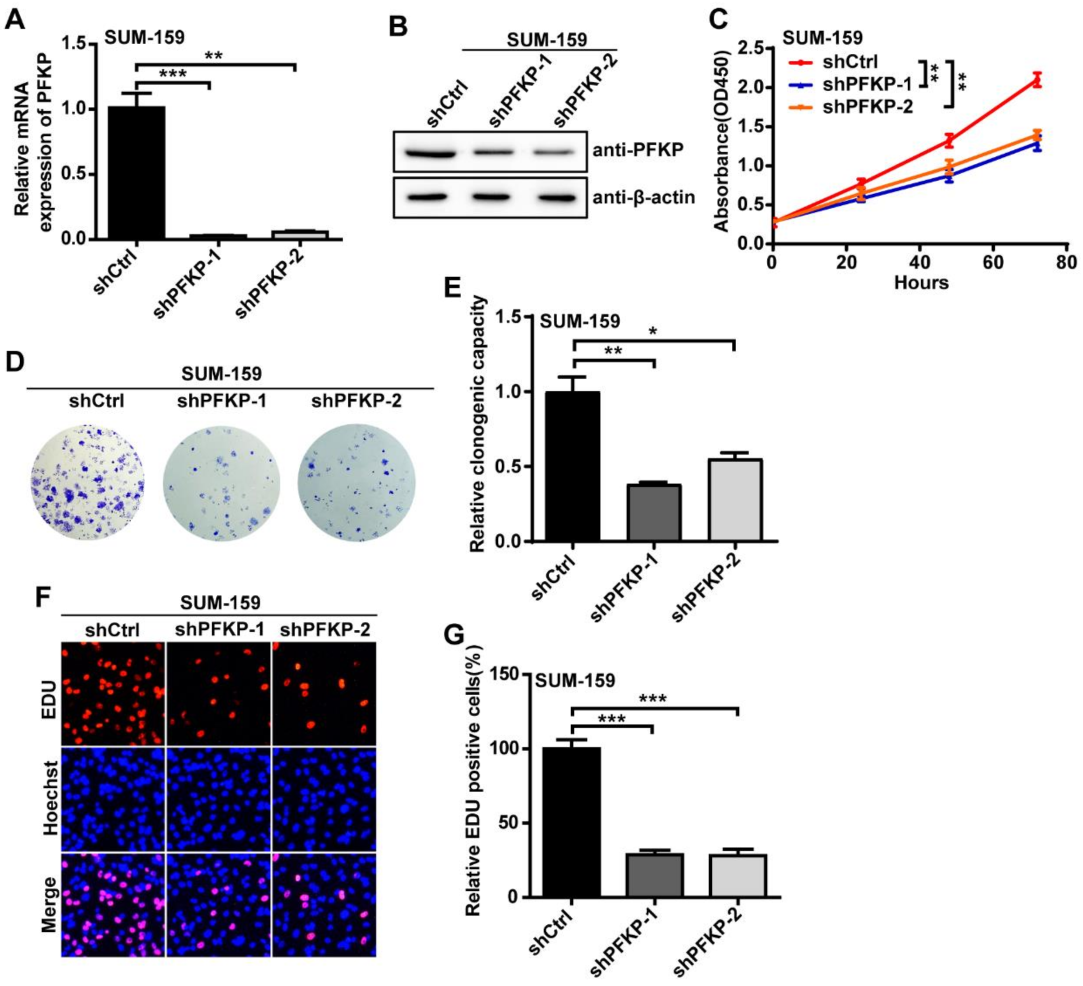

Supplementary Figure 1. (A, B) qRT-PCR **(A)** and western blot **(B)** analysis of PFKP expression with PFKP knockdown in SUM-159 cells. ****P* < 0.001, ***P* < 0.01. **(C)** CCK-8 assay determination of SUM-159 cell proliferation in response to PFKP knockdown. ***P* < 0.01. **(D, E)** Clone formation assay determination of SUM-159 cell proliferation in response to PFKP knockdown. ***P* < 0.01, **P* < 0.05. **(F, G)** EDU incorporation assay determination of SUM-159 cell proliferation in response to PFKP knockdown. ****P* < 0.001.

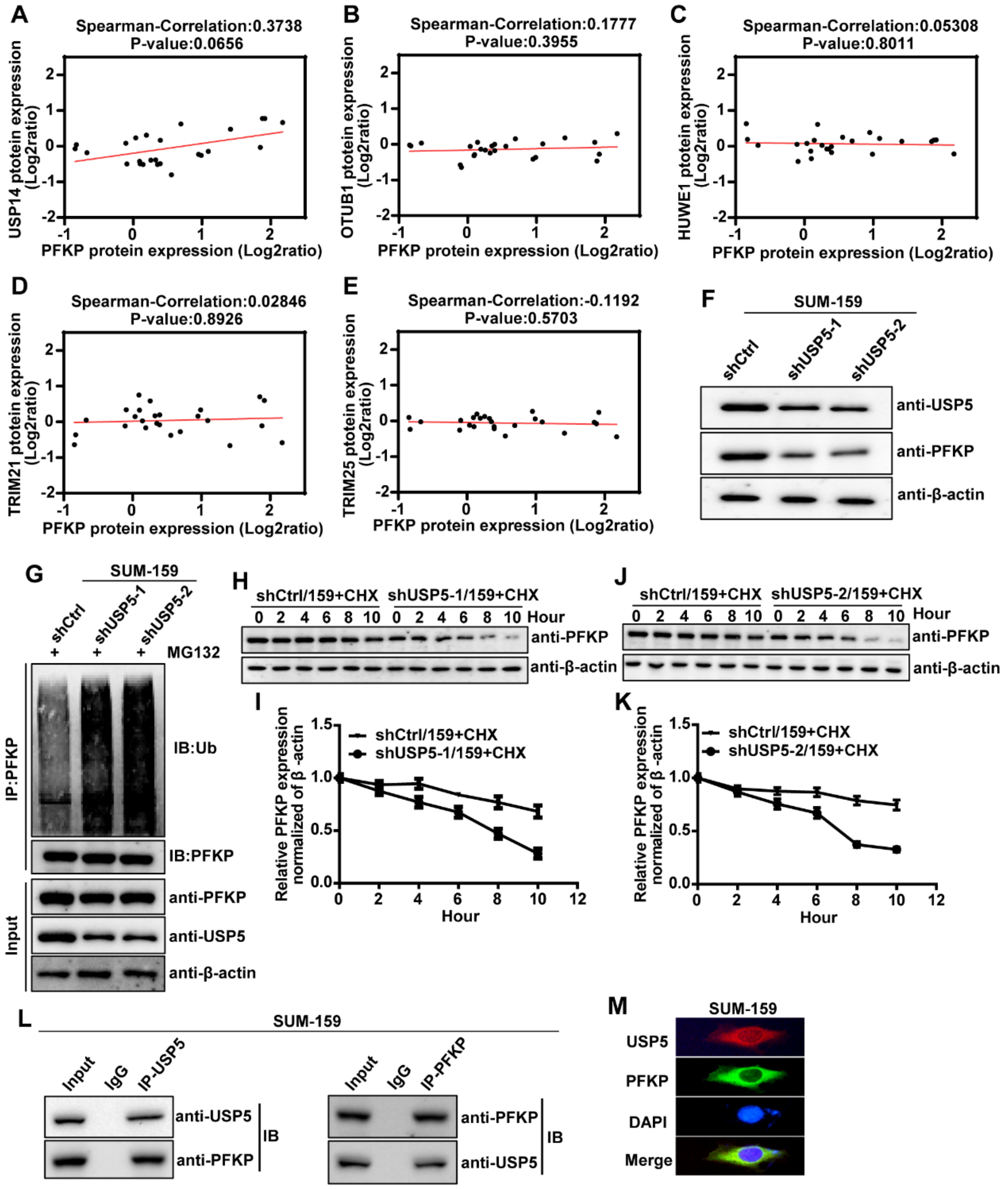

Supplementary Figure 2. (A-E) The correlation between USP14 **(A)**, OTUB1 **(B)**, HUWE1 **(C)**, TRIM21 **(D)**, TRIM25 **(E)** protein expression and PFKP protein expression in TNBC was analyzed by LinkedOmics platform. **(F)** The USP5 and PFKP protein expression in control and USP5 knockdown SUM-159 cells were analyzed by Western blot. **(G)** Co-IP analysis of PFKP protein ubiquitination in control and USP5 knockdown MDA-MB-231SUM-159 cells. **(H-K)** Control and USP5 knockdown SUM-159 cells were treated with CHX for the indicated time points and analyzed by Western blot **(H, J)**, quantification of PFKP protein levels normalized to β-actin in time after addition of CHX **(I, K)**. **(L)** The binding of USP5 to PFKP was detected by Co-IP assay in SUM-159 cells. **(M)** Immunofluorescence co-localization of USP5 and PFKP in SUM-159 cells.

Sup Figure 3

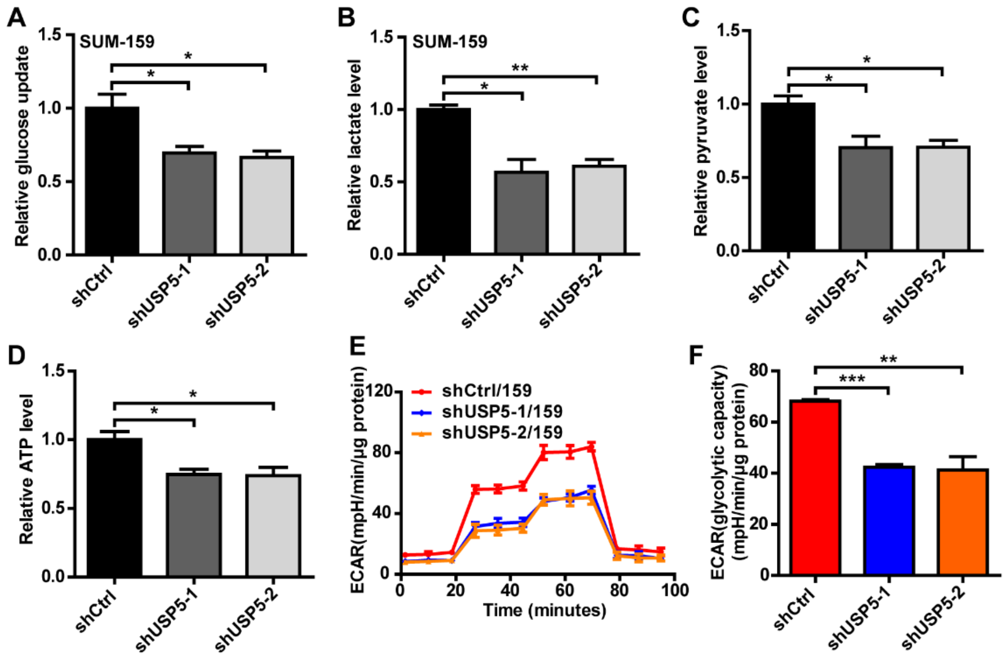

Supplementary Figure 3. (A-D) Glucose update **(A)**, lactate level **(B)**, pyruvate level **(C)**, and ATP level **(D)** were significantly decreased after USP5 knockdown in SUM-159 cells. **(E, F)** ECAR values **(E)** and calculated glycolytic capacity **(F)** of control and USP5 knockdown SUM-159 cells.

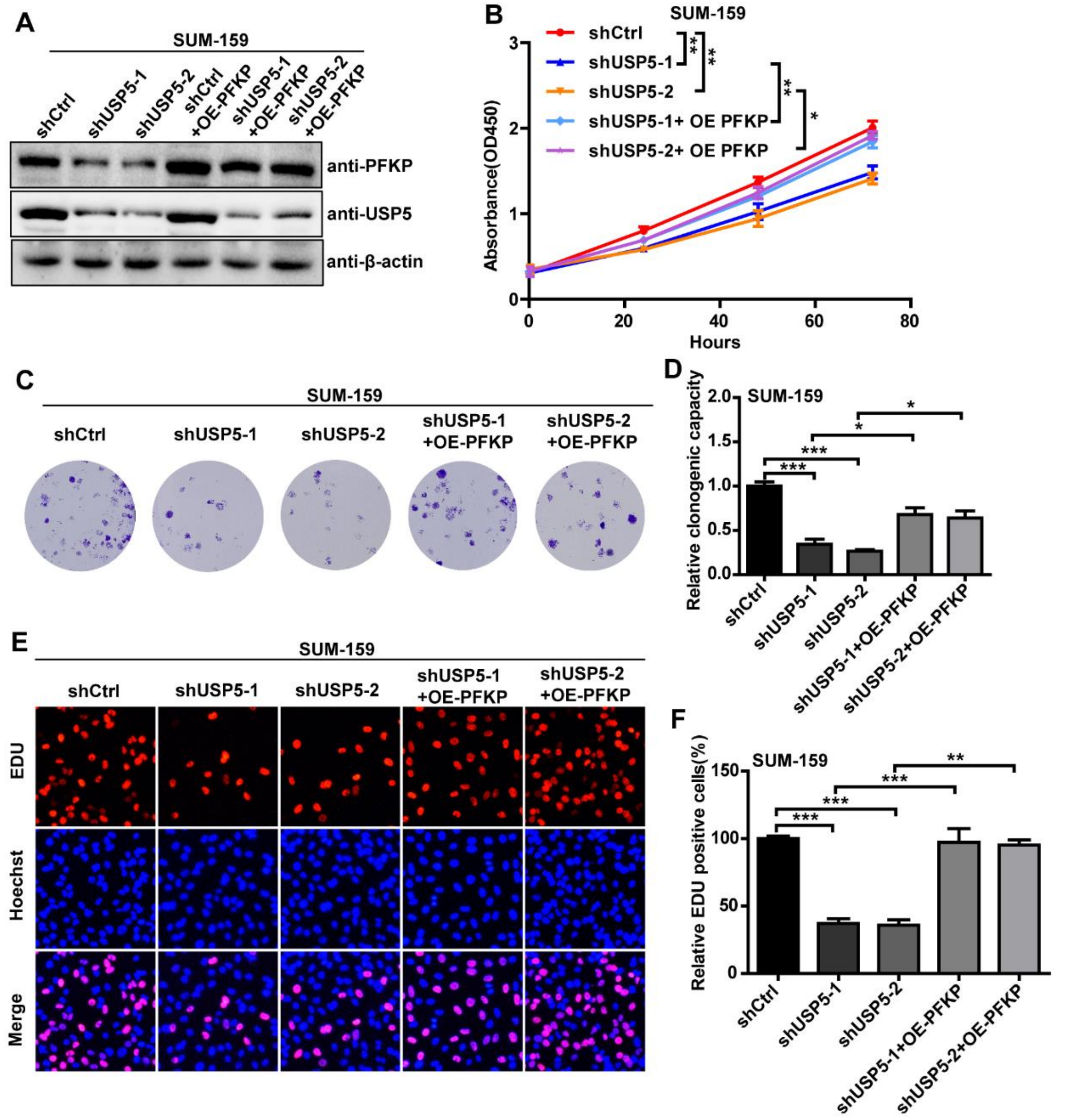

Supplementary Figure 4. (A) The USP5 and PFKP protein expression in control and USP5 knockdown SUM-159 cells in the presence or absence of overexpressed PFKP were analyzed by Western blot. **(B-F)** SUM-159 cell proliferation following USP5 knockdown in the presence or absence of overexpressed PFKP was evaluated using CCK8 assay **(B)**, Clone formation assay **(C, D)**, and EDU incorporation assay **(E, F)**. ****P* < 0.001, ***P* < 0.01.

Table S1. Primer used in this study.

Construction of PFKP expression vector	
PFKP forward	5’- ATGGACGCGGACGACTCCC -3’
PFKP reverse	5’- TCAGACACTCCAGGGCTGCACAT -3’
Construction of shRNA vector	
shPFKP-1	5’- GGGATGCTCAAGGTATGAA -3’
shPFKP-2	5’- GGCTGAAGGAGCAATTGAT -3’
shUSP5-1	5’- GCCTCTACATCTGTATGAA -3
shUSP5-2	5’- GCATCGACATGCTGAAGAT -3’
Quantitative RT-PCR	
PFKP forward	5’- GCATGGGTATCTACGTGGGG -3’
PFKP reverse	5’- CTCTGCGATGTTTGAGCCTC -3’
USP5 forward	5’- GCTGCTGTCAGTATTACCGAC -3’
USP5 reverse	5’- AAAGCCCAGAAACGTGTTCATA -3’
β-actin forward	5’- CATGTACGTTGCTATCCAGGC -3’
β-actin reverse	5’- CTCCTTAATGTCACGCACGAT -3’
